# A Question of Professional Conduct

**Published:** 1914-06-13

**Authors:** 


*To the Editor of* The Hospital.

### A Question of Professional Conduct.

*To the Editor of* The Hospital.

Sir,—I wonder if such conduct as the following would be considered professional.

A doctor was called in to an accouchement case. He had never seen the patient before, and within five minutes of his arrival she was under chloroform and instruments were used, though the patient had only been taken ill five hours previously, and according to the doctor himself the presentation was perfectly normal.

He had not been asked to take such steps beforehand when he was engaged, neither did he consult anyone, and when spoken to on the subject he simply replied, "What was the use of me waiting in your dining-room for an hour or two smoking cigarettes?" and he further added that the only danger he ran was of tearing the patient, which he did very slightly in this case. To me such conduct appears decidedly outrageous.—I am, Sir, yours faithfully,
X. Y. Z.

[If the facts are exactly as stated, the practitioner in question certainly behaved abominably. There are, however, two sides to every case, and he would possibly be able to justify his action if asked to.—Ed. The Hospital.]

